# Analysing the Predictors of Financial Stress and Financial Well-Being among the Bottom 40 Percent (B40) Households in Malaysia

**DOI:** 10.3390/ijerph191912490

**Published:** 2022-09-30

**Authors:** Maslina Mansor, Mohamad Fazli Sabri, Mustazar Mansur, Muslimah Ithnin, Amirah Shazana Magli, Abd Rahim Husniyah, Nurul Shahnaz Mahdzan, Mohd Amim Othman, Roza Hazli Zakaria, Nurulhuda Mohd Satar, Hawati Janor

**Affiliations:** 1 Faculty of Economics & Management, Universiti Kebangsaan Malaysia, Bangi 43600, Malaysia; 2 Department of Resource Management & Consumer Studies, Faculty of Human Ecology, Universiti Putra Malaysia, Serdang 43400, Malaysia; 3 Center for Sustainable & Inclusive Development Studies, Faculty of Economics & Management, Universiti Kebangsaan Malaysia, Bangi 43600, Malaysia; 4 Corporate Communication Unit, Ministry of Health Malaysia, Putrajaya 62000, Malaysia; 5 Department of Finance, Faculty of Business & Economics, Universiti Malaya, Kuala Lumpur 50603, Malaysia; 6 Department of Economics & Applied Statistics, Faculty of Business & Economics, Universiti Malaya, Kuala Lumpur 50603, Malaysia

**Keywords:** financial behaviour, financial knowledge, financial stress, financial vulnerability, financial well-being, locus of control

## Abstract

This paper aims to analyse factors affecting financial stress among the Bottom 40 Percent (B40) group of Malaysian households, reflecting overall financial well-being. Data were collected through questionnaires from 1008 respondents across five major regions in Malaysia. The data were analysed using Exploratory Factor Analysis (EFA) and Partial Least Squares-Structural Equation Modelling (PLS-SEM). This study provides evidence that financial behaviour, financial vulnerability (debt and income), and locus of control (luck and self-confidence) significantly affect financial stress among B40 households. The results show a significantly positive relationship between financial stress with financial vulnerability (debt and income) and locus of control (self-confidence). On the contrary, financial behaviour and locus of control (luck) show a significant negative relationship with financial stress. The result also indicates that financial stress affects financial well-being. Overall, the findings indicate that policy-makers should invent more effective and substantial stimulus packages or other measures to reduce the financial burden on B40 households. The findings could eventually provide insights for future research to delve into the social impact of financial stress. This study also has established a valid and reliable instrument to measure financial stress involving B40 households in Malaysia that eventually reflects the financial well-being of this group of people.

## 1. Introduction

Malaysia is performing well in the United Nations’ Sustainable Development Goals (SDGs) due to the remarkable low unemployment rate, infrastructural development, and high healthcare satisfaction [1]. Despite fluctuations in the global economy over the past decade, a rising gross domestic product (GDP) trend impacted positive growth from 2000 to 2019 [2,3]. The Department of Statistics Malaysia (DOSM) reported that the economy grew by 4.3% in 2019 and continued to expand in the first quarter of 2020 [4]. A nation’s economic strength is correlated with citizens’ earnings and wealth distribution, especially the level of poverty, the real per capita income, and the Gini coefficient [5].

However, 2020 and 2021 were hard years which threatened every country, including Malaysia. This is due to the global Coronavirus disease (COVID-19) pandemic, which has hampered the economy and inflicted distress across the globe [6]. The first Movement Control Order (MCO) was executed from 18 March 2020 until 12 May 2020; it entailed four phases to assist the Malaysian government’s efforts to keep the spread and mortality of COVID-19 under control [7,8]. While the measures have been critical for preventing the spread of COVID-19 in Malaysia, they also have devastating economic consequences. Malaysia is no exception, as the pandemic has significantly impacted the country’s macro-economy and the people’s economic welfare [9].

The economic slowdown triggered by COVID-19, which affected all industries, was Malaysia’s worst since the Asian financial crisis of 1998. As a result, the country’s growth market significantly weakened from the first quarter’s 0.7% year-on-year growth [10,11]. On a macro level, business, and service closures and MCO restrictions disproportionately impacted private consumption and business investment. According to the Ministry of Human Resources [12], about 100,000 Malaysians lost their jobs in various industries between March and December 2020. An additional 40,000 workers have been laid off by July 2021. As a result, Malaysia’s unemployment rate rose to 4.8 percent in the first quarter of 2021 from 3.2 percent in the fourth quarter of 2019 [13]. In addition, households’ average monthly gross income fell by 10.3% between 2020 and 2019, from RM7,901 in 2020 to RM7,089 in 2019. The decline was contributed by households or individuals who experienced loss or reduced income, reduced working hours and increased skill-related underemployment. Meanwhile, the median monthly household gross income registered a decline of −11.3% compared to 2019, with RM5,209 in 2020 compared to RM5,873 in 2019 [4].

The Malaysian government has classified household income into three categories: Top 20% (T20), with income earnings above RM10,971, Middle 40% (M40) with income ranging from RM4,851 to RM10,970 and lastly the Bottom 40% (B40) with earnings less than RM4,850 a month [4]. The World Bank analysis reported that low-income people are the most vulnerable to the pandemic. People with lower incomes are likely to suffer more from illnesses, diseases, and disabilities due to a lack of healthcare [14], while financial stress will contribute to a pattern of financial difficulties and decreased financial well-being. According to Suprapto [15], financial stress is when a person is concerned about their finances, such as pressure from debt or inability to meet obligations. The greater one’s financial stress, the worse their financial health. These two variables will always conflict, resulting in financial stress negatively impacting financial well-being.

If financial stress occurs among everyone, the situation worsens for the B40 group, which is predisposed to absolute poverty. Therefore, the study of financial stress has been acknowledged as crucial to overall well-being. Financial stress is considered to have a significant negative impact because those who experience it tend to be less capable of managing their financial well-being. Mokhtar & Abdul Rahman [16] found that financial stress, work environment, locus of control, and financial behaviour were associated with financial well-being. Magli et al. [17] analysed the impact of financial behaviour, financial stress, and locus of control as independent variables on the financial well-being of B40 households in Selangor. They discovered that financial behaviour and locus of control positively impact financial well-being by reducing financial stress. Mahdzan et al. [18] researched the subjective well-being of various income groups in Malaysia. They discovered substantial disparities in financial behaviour, internal locus of control, financial knowledge, and financial stress.

Despite its effects, little is known about the factors affecting financial stress among B40 households in Malaysia dealing with tough times in this pandemic. The COVID-19 pandemic and its subsequent economic impact are a double whammy that has seen policymakers and academics concerned about improving households’ financial well-being in order to increase their stability. Determining the severity of the impact on each group, particularly the most vulnerable, is challenging since previous research frequently failed to adequately account for households at the top and bottom of the income distribution [19]. Most studies addressed marginalised populations in general without focusing on a specific socio-economic income level [20,21,22]. Accordingly, the current situation has highlighted the importance of understanding and measuring financial stress, particularly in the most vulnerable demographic, the B40. Janssens et al. [23] highlighted that extensive analyses of the COVID-19 pandemic consequences for consumers and households are scarce, particularly in low- and middle-income nations. Hence, this gap motivated this study to examine the factors affecting financial stress among B40 households by tandem understanding the conditions during COVID-19.

Moreover, studying predictors of financial stress and financial well-being in low-income households is an essential component of household economics research. This preventative measure has far-reaching effects on the social and economic health of the household and its neighbourhood. The current situation has also highlighted the importance of understanding and measuring financial stress, particularly in the most vulnerable demographic, the B40. Janssens et al. [23] highlighted that extensive analyses of the COVID-19 pandemic consequences for consumers and households are scarce, particularly in low- and middle-income nations. Hence, this gap motivated this study to examine the factors affecting financial stress among B40 households by tandem understanding the conditions during COVID-19.

In addition, Malaysia and many other countries globally suffer the devastating impact of lost or reduced income, particularly on vulnerable low-income populations [24,25]. Theoretically, in the previous study across many countries, several factors affected financial well-being, such as financial behaviour, financial attitude, financial stress, personality traits, and financial literacy [26]. In practice, understanding the factors affecting financial well-being is very important [26,27]. Thus, by conducting this research, the finding not only benefits Malaysia but can also be referenced for other countries, as achieving and maintaining financial well-being is vital for individuals, families and the whole region. Output can also provide guidelines in policy-making to build stronger families that make stronger communities.

This study aimed to look into the causes that contribute to financial stress among B40 households by studying the selected variables: financial behaviour, financial knowledge, financial stress, financial vulnerability, and locus of control, and the relationship between financial stress and financial well-being of B40 households. Understanding all of the mentioned variables will facilitate policymakers in designing more effective and considerable stimulus packages to minimise the financial burden for the B40 households. Moreover, the findings could eventually provide insights for future research to delve into the social impact of financial stress among households in Malaysia.

## 2. Literature Review and Research Framework

### 2.1. Financial Well-Being

Research on consumer money management, spending, saving, and investment practices is a prominent field of study called “financial well-being” [26]. The capacity of a nation’s policymakers to raise its citizens’ financial well-being is frequently used to gauge economic growth [28]. Recent research has increasingly employed Consumer Financial Protection Bureau (CFPB) definitions of financial well-being [29,30,31].

Falahati et al. [32] describe financial well-being as a condition of being financially healthy, joyful, and worry-free, often based on an individual’s subjective assessment of their financial status. On the other hand, Brüggen et al. [33] conclude that the conceptualisation of financial well-being in the literature is fluid and ambiguous, resulting in numerous variations in its definition and assessment, which assert three clusters of financial well-being measurements: objective metrics, subjective measures, and a blend of objective and subjective data.

In the first cluster, Delafrooz et al. [34] utilise measures of objective financial well-being such as creditworthiness, the number of accessible emergency finances, the allocation of monthly credit card payments, monthly loan payments, monthly allocation of money for savings, and future retirement preparedness. In the second cluster, Kim et al. [35] quantify subjective financial well-being as views of debt status, ability to fulfil monthly expenses, and financial happiness. In addition, Mokhtar et al. [36] argue that one’s financial well-being may be influenced by the actions one takes to accumulate and protect wealth through financial and retirement planning with the use of readily available and accessible financial information.

The financial well-being concept was examined as a continuum of negative to positive feelings for certain financial conditions called the InCharge Financial Distress/Financial Well-being (IFDFW) Scale. Various IFDFW-based research has been undertaken in numerous countries, including Malaysia [16]. The third cluster of measures involved a combination of objective and subjective measures, as conducted by Xiao et al. [37], where the scholars combined the level of debt with perceived satisfaction with financial status.

### 2.2. Financial Stress

Financial stress is sometimes referred to as scarcity [38] or financial shock (Losada-Otálora & Alkire (née Nasr), 2019; Losada et al., 2018) [39,40]. The CFPB’s conceptualisations of financial stress, scarcity, and financial shock are related to the fact that consumers experience emotional and physical hardship due to insufficient money. Despite the extensive use of the term “financial stress” in the literature, the conceptual definition remains elusive [29,33]. Netemeyer et al. [29] defined and operationalised financial well-being by including the current money management stress model. The financial stress construct is utilised as an antecedent of financial well-being, a consequence of financial well-being, and even to define the financial well-being construct.

Financial stress is a complicated interaction between financial responsibilities and a shortage of funds. According to Steen and MacKenzie [41], financial stress raises the chance of hopelessness and damages one’s health and mental well-being. According to Kim and Garman [42], financial stress is a subjective assessment of one’s financial state, including perceived ability to pay expenses, satisfaction with one’s financial situation, amount of savings and investment, and debt anxiety. In its broadest sense, financial stress disturbs routine financial management, such as insufficiency, and frequently predicts psychological distress [43]. However, this association may be mitigated by social interactions such as family and community support, thereby minimising the negative impacts of financial stress on psychological well-being [44].

The results of a study conducted by Choi et al. [45] demonstrated a substantial association between job insecurity and financial stress and a partial mediation impact on financial well-being. In addition, income moderated the indirect effect of job insecurity on financial stress toward financial well-being. Whereas those with greater financial well-being were more likely to experience less financial stress, this association varied by income, with higher-income groups seeing a stronger correlation.

### 2.3. Financial Knowledge

Financial knowledge is the understanding of financial concepts and the skills and ability to manage money and make informed financial decisions. Hung et al. [46] define financial knowledge as fundamental knowledge of economic and financial concepts and the ability to effectively apply this knowledge and other financial skills to manage financial resources for a lifetime of financial security. In addition, financial knowledge is the capacity to comprehend and evaluate funding options, plan for the future, respond appropriately to situations [1], and address financial challenges or possibilities [47]. Depending on a person’s financial expertise, every person possesses financial knowledge, and financial contentment may also result from making sound financial decisions based on good financial knowledge.

Khan et al. [48] and Saurabh & Nandan [49] define financial knowledge as an individual’s fundamental comprehension of financial-related topics. Additionally, financial knowledge refers to the ability to comprehend, manage, and make judgments involving finances. In other words, it describes how individuals make, manage, and invest money, as well as how they contribute to the well-being of others. Specifically, financial literacy refers to the set of knowledge and skills that enables individuals to engage in and make productive financial decisions with all of their resources [50]. Previous research has demonstrated that individuals with a high level of financial knowledge have a higher financial well-being [49,51]. A wide range of skills and insights can be gained through financial knowledge.

In the marketing literature, the terms financial literacy and financial knowledge have been used interchangeably [29,52]. Warmath & Zimmerman [52] suggested a measure comprising financial knowledge, competence, and self-efficacy based on Bloom’s taxonomy of cognitive, affective, and psychometric learning theories in order to challenge this restrictive conception of financial literacy in terms of financial knowledge. In addition, the study demonstrates that financial literacy is an antecedent of financial well-being and that an increase in financial literacy will lead to an increase in financial well-being.

According to Sanderson et al. [53], financial knowledge, a fundamental component of the financial literacy model, influences a person’s ability to apply the knowledge and skills needed for appropriate financial decision-making toward efficient financial resource management. Hence, it has a significant positive impact on financial well-being because of financial knowledge and attitude, such as managing one’s finances and making sound financial decisions to improve one’s financial well-being. Based on the discussion in this article, this study hypothesises the following:

**Hypothesis** **1** **(H1).** *Financial knowledge has a positive correlation with financial stress in Malaysians’ B40 households.*

### 2.4. Financial Vulnerability

Financial vulnerability is defined as “the likelihood of a person experiencing financial difficulties.” Consequently, financial hardship is a condition of distress in which a person is unable to sustain their standard and quality of living [54]. The concept of financial vulnerability emphasises future direction (the potential for financial distress) while disregarding current living standards and money management. In the absence of empirical data, it is reasonable to presume that decreased financial vulnerability leads to increased financial well-being among individuals.

Financial vulnerability is generally linked to difficulties in meeting financial obligations and consumption and the risk of default. According to Lee & Sabri [55], financial vulnerability refers to financial insecurity or a scenario where financial risk and shock are present. In addition, scholars in health economics use the term “financial vulnerability” to describe households experiencing stressful conditions or severe health problems due to a high level of debt accumulation [56].

Households, particularly low-income households, are considered fragile and vulnerable if they cannot make ends meet, pay their monthly expenses, or have outstanding utility bills [57]. The most common indicators in determining financial vulnerability are ratios that reflect households’ ability to pay their obligations [58]. The absolute value of debt, however, is less useful in gauging household fragility unless it is compared to income or assets in the form of ratios: debt service-to-income ratio, unsecured debt service-to-income ratio, debt-to-income ratio, debt-to-asset ratio, and mortgage income gearing ratio [59]. Anderloni et al. [60] find substantial evidence that an increase in the debt service-to-income ratio increases the financial vulnerability of households. Hence, based on the prior discussion, this study hypothesises the following:

**Hypothesis** **2** **(H2).** *Financial vulnerability has a positive correlation with financial stress in Malaysian B40 households*.

### 2.5. Financial Behaviour

Jorgensen et al. [61] described financial behaviour in cash management, credit management, capital accumulation, and general management. Poor financial behaviour can result in economic difficulties [62]. On the other hand, financial knowledge may encourage positive financial behaviours, such as spending just on necessities, paying bills on time, maximising savings and investing, and acquiring personal and family health insurance [63,64]. Conversely, poor financial behaviours frequently lead to financial problems, with weak financial knowledge being a major contributor [51]. Studies have also proven that improved financial behaviour regarding economic management skills and knowledge will enable households to engage in healthier financial behaviour in daily life. Individuals’ financial well-being can be negatively impacted by a lack of financial knowledge in aspects including income management, retirement planning, savings, and contingency planning [51].

Financial behaviour can play a central role in triggering the financial stress of individuals, including those in households, societies, nations, and globally. According to Rajna et al. [65], financial behaviour can be viewed as a psychological tendency expressed when evaluating recommended financial management practices. According to Falahati et al. [32], financial behaviour refers to an individual’s capacity to manage their finances to achieve success in life. Halim & Astuti [66] define financial behaviour as a mental state, opinions, and evaluations regarding finances.

Several previous studies have also attempted to explain financial behaviour from various angles. Individual aspects of financial concepts, such as financial behaviour, are linked to financial management techniques and financial satisfaction [32,67,68]. According to Xiao et al. [69], financial behaviour influences financial happiness. Individuals who can effectively manage their finances, such as paying bills on time, being debt-free, and having savings, investments, and insurance, are more satisfied with their financial situation than those who cannot. It has a substantial positive impact because an intellectual approach to managing finances will improve financial well-being because people have goals to pursue [15]. The following hypotheses may be established as a result of the research conducted:

**Hypothesis** **3** **(H3).** *Financial behaviour has a positive correlation with financial stress in Malaysians’ B40 households*.

### 2.6. Locus of Control

The locus of control (LOC) has both an internal and an external dimension. Individuals with an internal locus of control believe that their actions govern future events, whereas those with an external locus of control believe that future events are determined by luck, chance, or fate and are beyond their control [61]. Regarding the financial LOC, individuals with a high internal LOC typically assume that their financial successes or difficulties are a result of their own efforts and work. Conversely, individuals with a high external LOC tend to attribute their financial success or failure to external variables beyond their control, such as bias, injustice, fate, luck, circumstance, or the faults of others.

Although LOC is not a new concept, studies on financial LOC are limited and scant [70]. Only in recent years, however, have social scientists made any serious attempt to integrate LOC into financial studies [71]. As a result, only a few notable scholars have investigated the relationship between LOC and financial norms such as financial well-being. Previous research has also discovered that LOC affects individuals’ financial or non-financial behaviours [70].

The locus of control defines human psychological characteristics [72]. One of the personality traits is the locus of control, defined as an individual’s belief in their ability to exert self-control or their mentality dictating their success or failure in life [73]. As a result, it can influence a person’s financial decisions. The desire to consume products not needed to achieve maximum satisfaction was linked to the individual’s locus of control over their consumption behaviour. The desire to consume a product is materially constrained by income. A person with a higher income is likely to be more financially responsible. As a result, one’s financial decisions were influenced by their earnings.

Robbins & Judge [74] defined locus of control as the degree to which a person felt confident that they determined their fate. There were two types of LOC: internal locus of control (more self-reliant on hope) and external locus of control (more relying on hope for others). The higher an individual’s internal locus of control, the greater their financial responsibility. This is because an individual is viewed as more capable of controlling them-self, managing finances, resisting the influence of others, being more motivated, and completing challenging tasks than those with a lower locus of control [72]. Thus, this causes a positive and negative relationship between locus of control and financial stress. As a result, the following hypothesis can be constructed:

**Hypothesis** **4** **(H4).** *Locus of control is positively related to financial well-being among Malaysian B40 households*.

### 2.7. Research Framework

**Figure 1:**
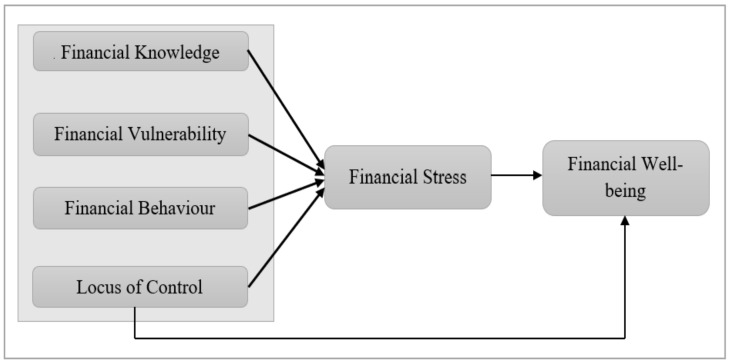
Research Framework.

Based on findings and discussion of the literature review, the research framework for this study is as shown in Figure 1:

## 3. Materials and Methods

### 3.1. Study Design and Data Sampling

**Figure 2:**
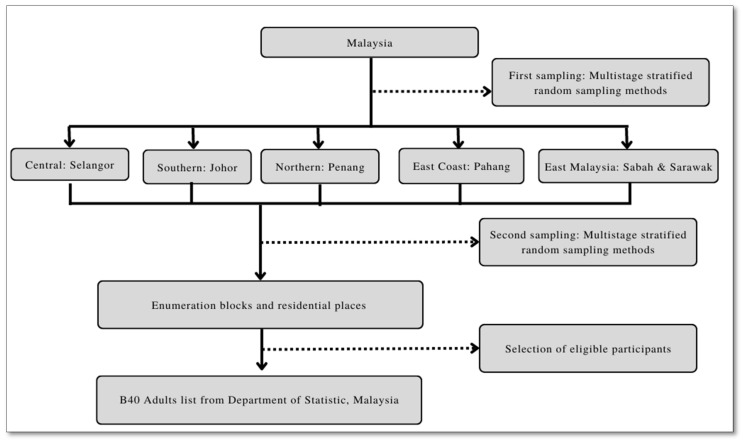
Flow chart of sampling method.

Malaysia has a population of 32.66 million people and 8.2 million houses as of 2020 [4]. This study employs a cross-sectional study using a quantitative approach, with all data gathered using a questionnaire survey. Two-stage sampling measures were employed. Figure 2 shows the sampling method of this research. 

The sampling frame for this study was obtained from the DOSM. In the first stage, a stratified sampling technique is conducted to validate the representativeness of households across five major regions in Malaysia (Central: Selangor, Southern: Johor, Northern: Penang, East Coast: Pahang, East Malaysia: Sabah & Sarawak). The states were chosen from different regions to reflect broader results and represent the B40 group nationwide. In the second stage, the B40 households were selected as respondents based on their income group classification, where monthly earnings were below RM4,850 [4].

The data collection period nationwide is from October 2020 to May 2021, involving 71 trained enumerators. One adult (aged 18 or older) per family represented the B40 households and responded to the survey questions. Malaysia has a population of 32.66 million people and 8.2 million houses as of 2020 [4]. On the other hand, the number of low-income B40 households increased from 405.4 thousand in 2019 to 639.8 thousand in 2020, as reported by the Department of Statistics Malaysia [4].

Low-income (B40) households were then identified using the National Household Sampling Frame (NHSF) list after consulting with the DOSM. After identifying missing data analysis, data cleaning, and straight-lining issues, 1008 usable data responses were acquired for further investigation. In addition, according to the sampling size calculation table by Krejcie & Morgan [75], the required sample size for a population equal to or larger than one million is approximately 384 samples with a 95% confidence interval and a 2.5% margin of error. This justification was supported by Hair et al. [76], Nunnaly [77] and Kline [78], whereby the sample size must be sufficiently large to ensure precise, statistically significant results. Henceforward, a deliberate sample method of 1008 is acceptable and adequate for this investigation, given that the sample represents a generalization of the low-income B40 population income group. Subsequently, a cross-sectional study using a quantitative approach was conducted in this study, with all data gathered using a questionnaire survey [1]. The sample’s representativeness has been analyzed, and thus it is possible to assess the generalizability of the results.

### 3.2. Study Instruments

The data for this study was collected through questionnaires. The questionnaire was divided into two sections. Section 1: respondent Background, which consists of respondents’ profiles, including questions on gender, age, race, marital status, education level, employment status before/during COVID-19, and household income. Meanwhile, Section 2 was divided into six sub-sections: financial behaviour, financial knowledge; financial stress; financial vulnerability; financial well-being; and locus of control. Financial knowledge-seeking information in the “yes” or “no” form. Conversely, in all of the other sub-sections (financial vulnerability, financial behaviour, financial stress, locus of control, and financial well-being), questions are in the Likert Scale form from the lowest score at 1, which indicates “never”, to the highest score at 4, which indicates “most frequent/always”. Each sub-section of financial stress, locus of control, and financial well-being contains eight questions each. At the same time, the rest of the sub-sections – financial knowledge, financial vulnerability, and financial behaviour – consisted of 10 questions in each sub-section.

Before the data collection, a series of pilot and pre-tests were undertaken to ensure the questionnaire’s validity and reliability. Henceforward, with the assistance of three academicians’ experts, a preliminary test/pre-test was conducted to determine the validity and consistency of the questionnaires.

All variables were subjected to content validation to ensure that the items were suitable for evaluating the target population’s understanding of financial well-being in the Malaysian context. Therefore, two experts in consumer finance and household financial management and one expert in consumption economics were asked to review and evaluate the items and rate the relevance of each item on a four-point Likert scale. A pilot test with 47 respondents was conducted in April 2020. The reliability was then determined using Cronbach’s alpha coefficient (α). The reliability coefficients for the four constructs ranged between 0.75 and 0.92, meeting the criteria of being greater than 0.7 [77]. As for the details, Cronbach’s alpha value for financial stress is 0.92, and Cronbach’s alpha values for constructs internal and external LOC are 0.78 and 0.75, respectively. The value of Cronbach’s alpha for financial behaviour was 0.88, financial vulnerability was 0.89, and Cronbach’s alpha for financial well-being was 0.84.

The original version of the questionnaire was written in Malay and edited by an expert for linguistic validation. The questionnaires were modified so respondents could comprehend them more efficiently by employing simple layman’s terms. The questionnaire was then pre-tested to confirm the questions’ meaning, language, flow, and comprehensibility. Finally, a few minor changes were made after refining the question statement.

For face validation, respondents were asked to complete the questionnaire and provide comments on the following items: the language and comprehension of the questions, the length of the questionnaire, the scoring method utilised, and the time required to complete the questionnaire. The feedback from participants was examined and considered, but it was not included in the data analysis [1].

### 3.3. Assessment of Data Distributions

Before analysing the model using Partial Least Squares-Structural Equation Modelling (PLS-SEM) techniques, it is essential to check data distribution to explore whether multivariate assumptions are met. Exploratory Factor Analysis (EFA) contains several tests: Kaiser-Meyer-Olkin (KMO), Bartlett’s test of sphericity, communalities, eigenvalues, and factor loadings.

KMO measures the sampling adequacy test, indicating that the data is adequate for exploratory factor analysis [46]. The sample is sufficient if the value of KMO is greater than 0.50 [79]. According to Hair et al. [76], Bartlett’s test of sphericity was required to assess the suitability of the data for factor analysis. Communalities indicate the amount of variance in each variable accounted for by the factor. The communality of each item should be 0.30 or higher to be retained. The item’s score will be deleted if it is below the minimum criterion. For eigenvalues, it should be greater than 1 and each factor should contribute a variance of 1. Eigenvalues are accumulated factors according to the amount of variance, and cumulative total variance should explain at least 50 percent. Factor loading for each item should be 0.50 or higher to be retained as an important item. Items were deleted if they failed to meet a minimum criterion (0.50 or higher).

### 3.4. Assessment of Common Method Biases (CMB)

Since we collected data through a questionnaire, common method bias (CMB) may be a potential concern. Therefore, we use Harman’s single-factor method and a marker variable assessment technique to assess CMB [80]. The Harman single-factor method is a post-hoc procedure conducted after data collection to check whether a single factor is accountable for variance in the data [81].

### 3.5. Measurement Model Analysis

SmartPLS is a software application for Partial Least Squares-Structural Equation Modelling (PLS-SEM) that consists of two components: a measurement model and structural model properties of data. The study’s measurement model was developed using Structural Equation Modeling (SEM), which is assessed through Exploratory Factor Analysis (EFA) and Confirmatory Factor Analysis (CFA). EFA is used to determine the constructed structure of the set of variables from Principal Component Analysis and Varimax. Meanwhile, confirmatory factor analysis (CFA) using SmartPLS.

The questionnaire survey data for this study were analysed through descriptive analysis using the PLS-SEM. Microsoft Excel was employed in data preparation. Under the measurement model, the first aspect of being observed is the outer loading. Outer loading between 0.40 and 0.70 can be accepted if other indicators/items with high loading can explain 50 percent (0.50) of the Average Variance Extracted (AVE) [82]. An indicator/item below 0.70 can be retained if the AVE value is above the threshold of 0.50 [83]. However, if AVE does not meet the threshold of 0.50, the reliability test can rely on CR due to the more accurate measurement of reliability [84].

The second step is to observe the internal consistency values of Cronbach’s Alpha (CA) and the Composite Reliability (CR). CR is optimal for PLS because it ranks variables according to their reliability, whereas CA is highly sensitive to the number of variables in each construct. The CA and CR are used to determine whether the sample is free of bias and whether or not the answers in their group are reliable. According to Hair et al. [48], a CA value of 0.60 and above is considered sufficient; ≥0.9 as excellent; between 0.8 and <0.90 as very good; between 0.7 and <0.8 as good; and between 0.6 and <0.7 as medium. CR values of between 0.70 and 0.90 are considered acceptable in exploratory research.

Next is the evaluation of the Average Variance Extracted (AVE). The AVE is the portion of the data (non-respective variables) explained by each of the constructs relative to their respective groups of variables or the average positive correlation between them and their constructs. When the AVEs are larger than 0.50, we can conclude that the model has converged to a satisfactory result [85]. However, if AVE does not meet the threshold of 0.50, the reliability test can rely on CR due to the more accurate measurement of reliability [84].

The fourth step is assessing the discriminant validity (DV) of the SEM, which indicates that the constructs or latent variables are independent. The main reason for DV is that items measuring different constructs or variables have poor relationships or low correlation. Failing to test this could lead to a chance that some items will have a good relationship with non-related items, indicating a lack of discriminant validity. With the guarantee of DV, the assessment of the measuring models was completed.

### 3.6. Structural Model Analysis

The next step is analysing the structural model with the first evaluation is Pearson’s Coefficient (R^2^). Pearson’s coefficient (R^2^) was performed using the SmartPLS to evaluate a Likert scale [86]. The R^2^ measures the proportional variance of the endogenous variables, as demonstrated by the structural model, indicating the quality of the adjusted model. The R^2^ measures the proportional variance of the endogenous variables, as demonstrated by the structural model, indicating the quality of the adjusted model. Cohen [87] states that R^2^ = 2% was defined as having a small effect, R2 = 13% as having a medium effect, and R^2^ = 26% as having a substantial impact on the social and behavioural sciences. A Variance Inflation Factor (VIF) was calculated to avoid a collinearity problem, and a VIF of 5 or lower needs to be obtained [76].

Then, the value of two other indicators of model adjustment quality is evaluated through Relevance or Predictive Validity (Q^2^) or Stone-Geisser indicator and Effect Size (f^2^) or Cohen’s Indicator [87]. The Q^2^ analyses how closely the model matches what was anticipated (or the adjusted model’s prediction quality or accuracy). As assessment criteria, values larger than zero must be acquired [76]. For example, a perfect model would have Q^2^ = 1 (which shows that the model reflects reality–without errors). The f^2^ is obtained by the inclusion and exclusion of model constructs. The small, medium, and large values are 0.02, 0.15, and 0.35, respectively [76]. In addition, f^2^ is evaluated based on the proportion of explained to unexplained information (f^2^ = R^2^/(1 − R^2^). Finally, the values of Q^2^ are obtained by reading the general redundancy of the model and f^2^ by reading the commonalities.

## 4. Results

### 4.1. Descriptive Analysis of the Participants

A total of 1088 respondents participated in this study, with 69.2% male and 30.8% female. As for age, 35.2% of the participants were in the age range of 51 and above. Furthermore, 73.7% of the respondents were married, followed by 15.2% with single status and widowed or divorced at 11.1%. The majority of the respondents were Malay (71.4%), followed by Indian (8.0%), Chinese (7.5%), Bumiputera Sabah (4.5%), Others (4.4%), and Bumiputera Sarawak (4.2%). The sample indicates that more than half (59.5%) of the respondents had a secondary level of education. In terms of employment, most of the respondents were employed before (82.5%) and during (81.9%) COVID-19. However, the percentage of working respondents slightly dropped during COVID-19 (0.6%). There were four groups of B40 income levels, with most respondents earning below RM2,500 (57.9%). The details of the respondents’ profiles were presented in Table 1.

### 4.2. Data Distribution and Common Methods Bias (CMB)

The unrotated principal component factor analysis (omitted for brevity) indicates a 20.988% total variance in Harman’s single-factor test, less than 50%, indicating that no single factor is loaded on all measures, which suggests there is no common method bias (CMB). An Exploratory Factor Analysis (EFA) with the Kaiser-Meyer-Olkin (KMO) test was used to identify whether the items in this questionnaire were suitable for the factor analysis and to test the sampling adequacy. The result of KMO is 0.894, Bartlett’s Test of Sphericity (20,864.705), degree of freedom (703), and significance (<0.001). The results show that it is acceptable to run a factor analysis, indicating that the correlation between the items is adequate for factor analysis. All of the VIF values (see Appendix A) were below 3.0 (VIF ranged from 1.085 to 1.389).

The principal component methods were employed with a cut-off eigenvalue of 1.00. The results show that five factors have been identified. All items’ value loadings are at an acceptable level of 0.50 or above (Appendix B). This signifies that all of the reasons are strongly correlated and exceed the threshold value of standardized factor loading. The cumulative total variance indicated 63.503 percent; therefore, the amount meets the criterion.

The first factor accounts for 13.162 percent of the total data variances associated with financial vulnerability (income). The second factor is financial vulnerability (debt), which explains 11.597 percent of the total data variances. Meanwhile, the third financial behaviour represents 10.284 percent of the total data variance. The fourth factor, locus of control (luck), explains 7.260 percent of the total data variances. The locus of control (self-confidence) represents 4.025 percent of the total data variances. Finally, financial stress and well-being are 8.151 and 6.138, respectively, (Appendix B). Meanwhile, the mean for financial knowledge with a yes/no response was 7.746 (SD = 1.900).

The outer loadings are the estimated relationships in a reflective measurement model, determining an item’s absolute contribution to its assigned construct. Overall, the outer loading is more than the minimum requirement of 0.40 (Appendix B). Therefore, item marks as ‘X’ indicate a value less than 0.4 (item financial well-being), which is unacceptable.

### 4.3. Measurement Model

Cronbach’s alpha (CA) tests were used to determine if multiple-question Likert scale surveys are reliable. CA indicates that all of the CA are above 0.60. All constructs show that CA values were between 0.78 and 1.00. This result shows that the internal consistency estimation of the data was adequate. The results revealed that the composite reliability (CR) values are greater than 0.70. Output of CR shows the value of CR ranges from 0.83 to 1.0, which exceeds 0.70, indicating that all items consistently measure their corresponding construct. The resultant average variance extracted (AVE) shows above 0.50 except for financial behaviour. Though financial behaviour values are below minimum criteria, the reliability test can rely on CR due to the more accurate reliability measurement (Appendix C).

There are eight different constructs assessed for DVL financial behaviour, financial knowledge, financial stress, financial vulnerability (debt), financial vulnerability (income), financial well-being, locus of control (luck), and locus of control (self-confidence) (Appendix D). The higher the record of correlation, meaning that the constructs might be related to each other, indicated a high correlation. The highest correlation constructs for financial stress were financial vulnerability-debt (0.662) and locus of control (self-confidence) (0.493). Meanwhile, the relationship between financial behaviour recorded the highest correlation with financial well-being (0.490).

### 4.4. Structural Model

After evaluating the measurement model, the next step is to test the structural confirmation of the hypotheses of relationships among the latent constructs. The structural model of PLS-SEM is assessed based on coefficients of determination (R^2^), predictive relevance (Q^2^), and significance of path coefficients. Table 2 shows that the R^2^ obtained for financial stress is 0.44, which means that 44% of the variance in financial stress was explained by financial behaviour, financial knowledge, financial vulnerability (debt), financial vulnerability (income), locus of control (luck), and locus of control (self-confidence). The R^2^ value of financial well-being is 0.011. The results for Q^2^ for each construct are 0.284 and 0.003. Both constructs yield Q^2^ greater than 0, thus showing the model is well-reconstructed. Moreover, all VIF values are less than 5, indicating that multicollinearity is very low.

Table 3 shows path coefficients (β) obtained for the structural model. Path coefficients (β) were based on probability values (*p*-values). The *p*-value ranges from 0 to 1, and a smaller *p*-value provides greater statistical data compatibility with the proposed research hypothesis. Five independent variables explained financial stress. A positive relationship was shown in financial vulnerability (debt) [β = 0.447, *t*-value = 14.984, *p*-value < 0.001], financial vulnerability (income) [β = 0.070, *t*-value = 2.489, *p*-value < 0.001] and locus of control (self-confidence) [β = 0.282, *t*-value = 9.070, *p*-value < 0.001] with financial stress. A negative relationship was shown between financial behaviour and financial stress (β = −0.149, *t*-value = 5.982, *p*-value < 0.001) and locus of control (luck) and financial stress (β = −0.086, *t*-value = 3.047, *p*-value < 0.05). Meanwhile, a negative relationship was shown between financial stress and financial well-being (β = −0.107, *t*-value = 2.054, *p*-value < 0.05).

On the other hand, Table 4 illustrates the indirect path coefficient between an independent variable and financial well-being. Financial behaviour, financial vulnerability (debt), locus of control (luck) and locus of control (self-confidence) were indirectly significant to financial stress and financial well-being. This means that if there are any changes in financial behaviour, financial vulnerability (debt), locus of control (luck) and locus of control (self-confidence), the effect on the financial stress and financial well-being is significant but indirect.

## 5. Discussion

The study findings offer insights into the influence of financial factors on the financial stress and financial well-being of Malaysian low-income households during the COVID-19 pandemic. This study investigated the critical antecedent of financial stress (FS) in Malaysian low-income (B40) households based on four independent variables, namely financial literacy (FL), financial behaviour (FB), financial stress (FS) and locus of control (LOC). The results showed that FB, FV (Debt), FV (Income), LOC (Luck) and LOC (Self-confidence) were significantly related to financial stress. FV (Debt), FV (Income) and LOC (Self-confidence) have a positive influence, whereas FB and LOC (Luck) have a negative impact on the financial stress of the B40. Additionally, a negative relationship was shown between financial stress and financial well-being. 

From the mean analysis for each item of the construct, the item financial vulnerability (debt), ‘I borrowed money from an unlicensed money lender’, recorded the lowest mean at 1.58. The record could reflect that most respondents believe they could be financially sound if they encountered a reasonable chance of changing their current financial state, such as a job promotion that increases their income. Even though they are dealing with financial difficulties, most respondents know the risk and consequences of borrowing from an unlicensed money lender. Hence, we assume respondents might avoid it and opt for another alternative method to support their living during tough times.

The financial vulnerability (income) with the highest means was ‘the income I received was not enough’ (2.48), while the lowest was ‘I don’t have cash for an emergency’ (2.36). According to Karlan et al. [88], saving can assist in smoothing consumption, making financially effective investments in human and corporate capital, and protecting against shocks for the poor, especially those in developing nations [88]. However, due to their low and unstable wages, saving money is challenging for the poor, and it is even more difficult to transform little savings into larger ones. Savings accounts with a financial institution may be helpful in this situation; hence, finding security and a reasonable return is of utmost importance to the poor who use savings products [89].

Furthermore, the highest agreement level for financial vulnerability (debt) is ‘I depend on side jobs or overtime to cover the cost of living’ (2.15). On the other hand, the lowest mean for financial vulnerability (income) is ‘I borrowed money from an unlicensed money lender’ (1.58). The highest agreed item is in line with the DOSM record that the number of employed people in 2020 dropped by 0.8 percent (116.7 thousand persons) to 15.0 million persons as against 15.1 million persons in 2019 [4].

The highest financial behaviour mean values were for items ‘I spend according to a weekly or monthly budget’ and ‘I scrutinise the price of the item carefully before buying it’, where the mean of both items was 3.03. The financial behaviour recorded the lowest agreement level in ‘I keep the purchase receipt’ (2.57). Respondents behave prudently towards their financial matters based on the highest agreed means. The situation is consistent with Perry & Morris’ [90] theory that financial behaviour manages an individual’s savings, expenses, and budget. We did assume that the lowest means did not show that the respondents were reluctant with their spending. However, it may reflect that the purchases are routine or involve petty purchases; thus, keeping the purchase receipt might be considered unnecessary for the respondents.

The highest mean for the locus of control (luck) was ‘I believe that opportunity is important in my life’ (3.15), while the lowest mean was ‘what happened to me was due to my efforts’ (2.93). The lowest agreement item might happen because of an unstable financial situation faced by the respondents during data collection due to external factors beyond the respondents’ control, such as COVID-19 and the current economy. The locus of control (self-confidence) highest agreement level is ‘I felt I had little influence over what happened to me’ (2.57), and the lowest mean is ‘I feel like I have no control over the family income’ (2.33). Respondents concurred that they had limited control over their situations. We presume they felt this way due to circumstances beyond their control, and they are merely holding on, especially regarding household income.

The highest item for financial stress was ‘I couldn’t sleep because I was worried about paying the bill’ (2.22). Financial stress recorded the lowest agreement level in ‘I have high blood pressure due to financial difficulties’ (1.93). According to Magli et al. [17], financial stress has a detrimental effect on an individual’s financial status. The deterioration of these qualities will have a negative impact on an individual’s financial well-being. A study among healthcare professionals demonstrated that financial stress is crucial in explaining financial well-being. The record for the highest agreement on the item is in line with the scholars. If the anxiety persists for an extended period, this could result in a health problem. Nevertheless, the lowest recorded item shows that respondents did not have hypertension because of financial hardship. The highest for financial well-being means is ‘I have at least three months of income savings for emergency purposes’ (2.56). The lowest mean for financial well-being is ‘I can afford to pay utility bills’ (1.96). According to Delafrooz et al. [34], financial well-being has evolved from simple happiness or general contentment with one’s material or financial state to a more sophisticated assessment that combines the material and non-material components of an individual’s financial situation. Financial stress explains 1.1% of the variance in financial well-being. This might happen because family well-being is considered to be a multi-dimensional concept that incorporates family relationships, the family’s economic situation, health and safety, community relationships, housing, and the environment, as well as religion and spirituality [91].

Anyone could experience financial stress, but it may occur more often in low-income households. As the *t*-statistic and 95% confidence interval indicates, all path coefficients are significant at the 0.01 level except for the relationship between financial stress and financial knowledge (*p*-value = 0.828). According to research by Courchane [92], self-assessed knowledge is one of the most influential determinants of financial behaviour. However, research demonstrates that individuals do not always have a complete sense of financial knowledge. Therefore, we presume that the respondents may have limited financial knowledge and will only use it when necessary. Furthermore, financial knowledge may not directly influence financial stress and might need to be examined in different observations. According to Sabri, et al. [93], financial well-being may be achieved by the adoption of appropriate behaviors that are influenced by an individual’s unique set of values, beliefs, skills, and personal experiences. In fact, a lack of financial knowledge or financial illiteracy is one of the causes of financial stress that diminish financial well-being [94,95].

Financial behaviour shows a negative relationship with financial stress, which means a higher level of financial behaviour will cause a lower level of financial stress, and vice-versa. The locus of control (luck) and financial stress show a negative relationship. It is significant, which means a lower level of locus of control (luck) will cause a higher level of financial stress which is in line with the study by Rajna et al. [65]. They assert that the better a person’s financial perspective is, the more aware they are of the need to save money, influencing their financial behaviour.

On the contrary, financial vulnerability (debt), financial vulnerability (income), and locus of control (self-confidence) show a positive relationship with financial stress. This indicates that a higher level of any of these variables will cause a higher level of financial stress. For instance, a higher level of financial vulnerability (debt) will trigger a higher level of financial stress and vice-versa. Additionally, a lower level of financial vulnerability (income) will lower financial stress. The situation is a typical circumstance where financial stress will trigger once the household is exposed to financial vulnerability, either debt or income. According to Turunen & Hiilamo [56], financial vulnerability describes households experiencing stressful conditions or severe health problems due to high debt accumulation in health economics. Research by Rajna et al. [65] on the influence of financial socialisation, financial behaviour, locus of control, and financial stress on young adults’ financial vulnerability has shown that financial stress positively predicts financial vulnerability. The study showed that a higher level of financial behaviour, and locus of control would contribute to lower levels of financial vulnerability.

Meanwhile, the finding shows that when financial stress rises, financial well-being will decline and vice-versa. This is consistent with the findings by Magli et al. [95], indicating that financial stress negatively impacts a household’s financial status and well-being. In a previous study, the scholar examined the roles of locus of control and parental financial communication in adult financial behaviour in the United States. The study, among others, suggested that adults who received better financial instruction engaged in more sound financial behaviour [61]. Moreover, a study conducted in India on the relationships between financial knowledge, socialization, and financial satisfaction has found that financial risk attitude and financial behaviour mediate the relationship between financial knowledge and financial satisfaction [49]. Meanwhile, another study in Indonesia on the influence of financial literacy, financial socialization, financial attitude, and financial confidence on financial well-being showed that financial literacy, financial socialization, financial attitude, financial confidence, and financial behaviour positively affect financial well-being [96].

### 5.1. Suggestion

Financial education is not a formal education that can only be taught in school. This skill can be cultivated at a younger age; thus, financial management knowledge can begin at home. Parents can play an important role in instilling financial knowledge in their children. In line with this, a well-planned financial education is critical for controlling financial decisions. Financial education should be introduced as early as possible and taught in schools as part of the syllabus. Incorporating financial education into the school curriculum is a fair and effective policy tool. The recommendation also echoes the Organisation for Economic Co-Operation and Development (OECD) [97] suggestion that this knowledge is instilled in the younger generation by incorporating it into the school curriculum. Hence, incorporating financial knowledge into the school curriculum could act as a policymaker’s long-term investment in human capital.

According to the literature, individuals who practice prudent financial behaviour will have better financial circumstances and improve their financial well-being. As a result, a financial institution such as Credit Counselling & Management Agency (AKPK) should step up its efforts and functions to create a financially literate society, particularly among the B40. Effective programmes could reach out to the target group and keep them from experiencing financial difficulties, which could lead to financial stress. In addition, financial advisors and consultants may create training programs or consult on financial stress management.

Financial education can make a difference by providing young people with the knowledge, skills, and confidence they need to take charge of their lives and create a more secure future for themselves and their families. Poor financial decisions can have long-term consequences for individuals, families, and society [98]. Therefore, they need to have and use their financial knowledge to avoid financial trouble. Furthermore, applying financial knowledge to young married couples will lay a stronger foundation for a better understanding of the orientation of financial knowledge that will be useful later in life. As a result, the relevant government department or agencies may need to make an effort by offering a mandatory introductory financial course to the young couple before registering their marriage.

Moreover, government intervention is required to ensure that the heads of B40 households can earn a decent living wage. Furthermore, a supportive economic policy is necessary to assist them in managing their financial well-being through better employment schemes, education, or training, for example, through Technical and Vocational Education and Training (TVET).

Henceforward, in the long-term, desirable government intervention would encourage responsible practices such as prudent use of consumer loans, debt restructuring, financial education, and debt advisory services to aid households in improving their financial management. As a result, this intervention could help to ensure that the heads of B40 households earn a living income.

### 5.2. Limitations

There are several limitations to this study. Firstly, reporting biases during the data collection can occur as the data was collected using self-reported questionnaires. Secondly, comparisons between the findings of this study and other analyses should be interpreted with caution due to differences in methodology and context. Future research could address this limitation by including states other than the region chosen, as the results could be biased and not representative of the population.

## 6. Conclusions

Financial stress refers to the specific emotional discomfort associated with money concerns. This study examines the factors contributing to financial stress among B40 households and reveals their overall financial health. The research presents various hypotheses. The results indicated that all of the hypotheses were accepted, except for the first, which stated: “Financial knowledge influences financial stress”; this is because its *p*-value was greater than 0.05.

Accordingly, it was determined that financial stress was positively correlated with financial vulnerability (debt and income) and locus of control (self-confidence). In contrast, financial stress correlates negatively with financial behaviour and locus of control (luck). In addition, financial behaviour, financial susceptibility (debt and income), and locus of control (luck and self-confidence) were significant elements influencing the financial well-being of B40 households.

Even if the Malaysian government has already loosened restrictions in response to the opening of economic sectors, the COVID-19 pandemic significantly influences the financial well-being of B40 communities in Malaysia. Household financial well-being is the capacity of a household to meet ongoing financial obligations, remain resilient to income shocks, achieve future financial goals, and make financial decisions that improve its quality of life. This is because a person facing financial stress will have difficulties experiencing and executing financial well-being.

It is proposed that future research studies utilise observations, qualitative designs, and empirical studies. The findings would provide evidence and a more precise understanding that the financial assistance provided by governments throughout the pandemic period helped lower financial stress and enhance the financial well-being of the B40. Theoretically, financial well-being has been acknowledged as a factor that can increase the socio-economic level of households. Significantly, the results of this study demonstrate the association between financial parameters, financial stress, and well-being, which can inform future interventions that target these aspects. In addition, the implementation of the relevant government and institutional interventions or programmes for the B40 populations, including the development of more effective and substantial measures and policy, could ease or reduce financial stress.

The findings of this study contribute to our understanding of the relationship between financial stress and well-being. This model can be applied not just to the Malaysian population but also to other nations in order to comprehend their populations better. The greater the household’s financial health, the lower the incidence of financial stress, and the greater the individual’s financial well-being.

## Figures and Tables

**Table 1:** Respondents’ profile (N = 1008).

Variables		No	(%)
Gender	Male	698	69.2
	Female	310	30.8
Age (Years)	21–30	205	20.3
	31–40	207	20.5
	41–50	241	23.9
	51–60	229	22.7
	61–70	102	10.1
Ethnicity	Malay	720	71.4
	Chinese	76	7.5
	Indian	81	8.0
	Bumiputera Sabah	45	4.5
	Bumiputera Sarawak	42	4.2
	Others	44	4.4
Marriage	Single	153	15.2
	Married	743	73.7
	Widowed/Divorced	112	11.1
Education	No formal education/Pre-school	41	4.1
	Primary	62	6.2
	Secondary	600	59.5
	Tertiary	277	27.5
	Others	28	2.8
Working status before COVID-19	Yes	832	82.5
	No	176	17.5
Working status during COVID-19	Yes	826	81.9
	No	182	18.1
Monthly household income (RM)	2500 or less	584	57.9
	2501–3170	185	18.4
	3171–3970	119	11.8
	3091–4850	120	11.9

**Table 2:** R Square and Q Square.

Construct	R Square	R Square Adjusted	Q Square
Financial Stress	0.441	0.438	0.284
Financial Well-Being	0.011	0.010	0.003

**Table 3:** Path Coefficients.

Relationship	Beta	SD	*t* Value	*p*-Value	f^2^	VIF
FB → FS	−0.149	0.025	5.982	0.000	0.035	1.132
FK →FS	−0.006	0.028	0.218	0.828	0.000	1.109
FV (Debt) → FS	0.447	0.030	14.984	0.000	0.240	1.484
FV (Income) → FS	0.070	0.028	2.489	0.013	0.007	1.323
LOC (Luck) → FS	−0.086	0.028	3.047	0.002	0.011	1.205
LOC (Self-confidence) → FS	0.282	0.031	9.070	0.000	0.112	1.272
FS → FWB	−0.107	0.052	2.054	0.040	0.012	

SD = Standard Deviation; VIF = Collinearity Statistics; FB = Financial behaviour; FL = Financial literacy; FS = Financial stress; FWB = Financial well-being; LOC = Locus of control.

**Table 4:** Indirect Path Coefficients.

Relationship	Beta	SD	*t* Value	*p*-Value
FB → FS → FWB	0.016	0.008	1.923	0.055
FK → FS → FWB	0.001	0.004	0.179	0.858
FV (Debt) → FS → FWB	−0.048	0.024	2.018	0.044
FV (Income) → FS → FWB	−0.007	0.005	1.403	0.161
LOC (Luck) → FS → FWB	0.009	0.005	1.829	0.068
LOC → FS → FWB	−0.030	0.015	2.018	0.044

SD = Standard Deviation; VIF = Collinearity Statistics; FB = Financial behaviour; FL = Financial literacy; FS = Financial stress; FWB = Financial well-being; LOC = Locus of control.

## Data Availability

All relevant data are within the paper.

## Appendix A

**Table A1:** Collinearity Statistics (VIF).

Construct	Random
Financial Behaviour	1.088
Financial Knowledge	1.088
Financial Stress	1.389
Financial Vulnerability (Debt)	1.380
Financial Vulnerability (Income)	1.143
Locus of control (Luck)	1.121
Locus of Control (Self-confidence)	1.085

## Appendix B

**Table A2:** Mean, Exploratory Factor Analysis (EFA) and Outer Loading.

Constructs/Items	EFA Loading	Communalities	Variance Explained	Mean	Outer Loading
**Financial Vulnerability (Income):**			13.162		
I can’t make savings every month.	0.788	0.669		2.416	0.790
I don’t have cash for an emergency.	0.778	0.687		2.364	0.819
The income I received was not enough.	0.766	0.670		2.482	0.826
The job I was in did not provide a stable income.	0.637	0.531		2.427	0.759
**Financial Vulnerability (Debt):**			11.597		
I depend on side jobs or overtime to cover the cost of living.	0.515	0.438		2.159	0.652
I borrow to buy daily necessities (basic necessities).	0.729	0.729		1.817	0.866
I borrowed money from an unlicensed money lender.	0.788	0.749		1.588	0.825
I have an outstanding monthly loan.	0.776	0.732		1.828	0.850
I couldn’t afford to make expenses at the end of the month because I ran out of money.	0.682	0.673		2.062	0.804
**Financial Behaviour:**			10.284		
I spend according to a weekly or monthly budget.	0.706	0.532		3.032	0.452
I keep track of where my money is spent.	0.674	0.548		2.692	0.700
I set aside money for emergency expenses.	0.759	0.658		2.887	0.649
I save to meet personal/family financial goals.	0.718	0.595		2.918	0.593
I keep the purchase receipt.	0.646	0.545		2.577	0.839
I made the minimum payment for the loan made.	0.582	0.476		2.625	0.814
I scrutinise the price of the item carefully before buying it.	0.587	0.524		3.032	0.472
I have long-term financial goals and strive to achieve them.	0.631	0.518		2.938	0.565
**Locus of control (Luck):**			7.260		
What happened to me was due to my efforts.	0.729	0.648		2.930	0.943
My financial situation depends on my ability to control the situation.	0.800	0.733		3.055	0.849
I believe that opportunity is important in my life.	0.760	0.632		3.155	0.544
**Locus of Control (Self-confidence):**			6.913		
I feel it is not wise to plan far ahead because many things will change.	0.653	0.559		2.457	0.719
I felt I had little influence over what happened to me.	0.704	0.593		2.573	0.710
I feel like I have no control over the life I live.	0.800	0.725		2.359	0.873
I feel like I have no control over the family income.	0.776	0.697		2.337	0.858
**Financial Stress:**			8.151		
I couldn’t sleep because I was worried about paying the bill.	0.703	0.538		2.229	0.687
I am getting depressed and restless with my current financial situation.	0.742	0.652		2.152	0.779
I can’t afford (financially) to see a doctor when I’m sick.	0.804	0.734		2.004	0.857
I can’t afford (financially) to get healthier food.	0.813	0.755		2.019	0.872
I have high blood pressure due to financial difficulties.	0.786	0.747		1.932	0.861
I’m worried about medical expenses.	0.794	0.696		2.176	0.813
I became depressed as a result of thinking about finances.	0.774	0.692		2.045	0.839
**Financial Well-Being:**			6.138		
I can afford to buy the stuff I want.	0.752	0.618		2.166	0.549
I can afford to pay utility bills.	0.590	0.527		1.967	X
I have extra money at the end of the month.	0.770	0.686		2.346	0.662
I can achieve short-term financial goals (examples: buying furniture and electrical goods).	0.770	0.666		2.347	0.800
I can achieve long-term financial goals (for example: buying a house).	0.784	0.651		2.491	0.859
I have at least RM1,000 for emergency expenses.	0.717	0.623		2.457	0.824
I have at least three months of income savings for emergency purposes.	0.783	0.687		2.560	0.859

Extraction Method: Principal Component Analysis. Rotation Method: Varimax with Kaiser Normalization. Kaiser-Meyer-Olkin Measure of Sampling Adequacy (0.894), Bartlett’s Test of Sphericity (20864.705), Degree of freedom (703), significant (0.000). Total variance explained: 63.504.

## Appendix C

**Table A3:** Construct Reliability and Validity.

Constructs	Cronbach’s Alpha	Composite Reliability	Average Variance Extracted (AVE)
Financial Behaviour	0.846	0.848	0.422
Financial Knowledge	1.000	1.000	1.000
Financial Stress	0.916	0.933	0.668
Financial Vulnerability (Debt)	0.860	0.900	0.645
Financial Vulnerability (Income)	0.811	0.876	0.638
Financial Well-Being	0.889	0.894	0.589
Locus of control (Luck)	0.789	0.833	0.635
Locus of Control (Self-confidence)	0.805	0.871	0.630

## Appendix D

**Table A4:** Discriminant Validity- Heterotrait-Monotrait Ratio (HTMT).

Constructs		FB	FK	FS	FV-Debt	FV-Income	FWB	LC-Luck	LC-Self Confidence
**1**	**FB**								
**2**	**FK**	0.187							
**3**	**FS**	0.191	0.118						
**4**	**FV-Debt**	0.148	0.145	0.662					
**5**	**FV-Income**	0.126	0.036	0.364	0.567				
**6**	**FWB**	0.490	0.205	0.101	0.141	0.323			
**7**	**LC-Luck**	0.368	0.216	0.088	0.102	0.106	0.351		
**8**	**LC-Self confidence**	0.140	0.205	0.493	0.380	0.171	0.190	0.411	

FB: Financial Behaviour, FK: Financial Knowledge, FS: Financial Stress, FV: Financial Vulnerability, FWB: Financial Well-Being, LC: Locus of control.
